# Pattern of Breast Cancer Distribution in Ghana: A Survey to Enhance Early Detection, Diagnosis, and Treatment

**DOI:** 10.1155/2016/3645308

**Published:** 2016-08-18

**Authors:** Frank Naku Ghartey Jnr, Akwasi Anyanful, Sebastian Eliason, Saanid Mohammed Adamu, Samuel Debrah

**Affiliations:** ^1^Department of Chemical Pathology, School of Medical Sciences, College of Health and Allied Sciences, University of Cape Coast, Cape Coast, Ghana; ^2^Department of Medical Biochemistry, School of Medical Sciences, College of Health and Allied Sciences, University of Cape Coast, Cape Coast, Ghana; ^3^Department of Community Medicine, School of Medical Sciences, College of Health and Allied Sciences, University of Cape Coast, Cape Coast, Ghana; ^4^Department of Surgery, School of Medical Sciences, College of Health and Allied Sciences, University of Cape Coast, Cape Coast, Ghana

## Abstract

*Background*. Nearly 70% of women diagnosed with breast cancer in Ghana are in advanced stages of the disease due especially to low awareness, resulting in limited treatment success and high death rate. With limited epidemiological studies on breast cancer in Ghana, the aim of this study is to assess and understand the pattern of breast cancer distribution for enhancing early detection and treatment.* Methods*. We randomly selected and screened 3000 women for clinical palpable breast lumps and used univariate and bivariate analysis for description and exploration of variables, respectively, in relation to incidence of breast cancer.* Results*. We diagnosed 23 (0.76%) breast cancer cases out of 194 (6.46%) participants with clinically palpable breast lumps. Seventeen out of these 23 (0.56%) were premenopausal (<46.6 years) with 7 (0.23%) being below 35 years. With an overall breast cancer incidence of 0.76% in this study, our observation that about 30% of these cancer cases were below 35 years may indicate a relative possible shift of cancer burden to women in their early thirties in Ghana, compared to Western countries.* Conclusion*. These results suggest an age adjustment for breast cancer screening to early twenties for Ghanaian women and the need for a nationwide breast cancer screening to understand completely the pattern of breast cancer distribution in Ghana.

## 1. Introduction

Cancer of the breast is the most common cancer in both developing and developed countries and is responsible for over one million in an estimated ten million neoplasms diagnosed in both sexes worldwide [1]. Breast cancer is also the primary cause of cancer death among women globally, responsible for about 425,000 deaths in 2010 [2]. In 2012, nearly 1.7 million new cases of breast cancer were diagnosed worldwide (second most common cancer overall after cervical cancer), representing about 12% of all new cancer cases and 25% of all cancers in women [3]. This is an increase from 2010 when 1.6 million new cases of breast cancer were diagnosed [2]. Increasing incidence of breast cancer is also being reported for developing countries in Africa, in contrast to previous reports [4]. For example, a 2011 American Cancer Society study reports that “in several sub-Saharan African countries, breast cancer has now become the most commonly diagnosed cancer in women, a shift from previous decades in which cervical cancer was the most commonly diagnosed cancer [5].” This observation is consistent with studies in Cote d'Ivoire [6], Uganda [7, 8], Nigeria [9], and Ghana [10]. Though these increases are still low compared to developed countries, higher mortality rates accounting for 75% of total deaths from the disease are observed in developing countries [11]. This is because priority is given to the control of communicable diseases in developing countries thus relegating cancer screening and care to the background resulting in paucity of reliable and quality cancer data including breast in many developing countries [12].

Despite these challenges, several general aspects of breast cancer burden in African women have been characterized including lower lifetime risk of developing breast cancer comparatively [4, 13] and most likely occurring in premenopausal women [4, 14] with peak incidences approximately 10 years earlier than the Western counterparts [4, 13]. Other reports indicate that breast cancers are typically presented late at treatment centres [15, 16] resulting in significantly larger tumours [17] that are difficult to treat. Low awareness and unavailable mass screening programs, inaccessible specialized treatment centres, and sociocultural beliefs even among educated African women and females in the health profession have been proposed as possible reasons for the late presentation. For example, a cross-sectional survey of 204 Nigerian nurses revealed that 34% were unfamiliar with breast cancer risk assessment and most believed they were not at risk [18]. In a second survey among school teachers, only 53% realized that the presence of lump(s) in the breast is a significant symptom of breast cancer even though 85% of respondents knew that breast cancer was a serious disease [19]. Outside urban areas, awareness and understanding of breast cancer is relatively low in the general population and supernatural phenomenon is the most common reason cited for the cause of breast cancer [20].

In Ghana, breast cancer is a major public health problem and the most common type of cancer among women in terms of mortality and incidence [21]. The incidence of breast cancer is also increasing in Ghana because data derived from the Korle Bu Teaching Hospital Cancer Register from 1972 through 1975 shows that breast cancer accounted for 7.5% of all cancers in Ghana and was the fourth most common cancer after liver carcinoma, cervix cancer, and Burkitt lymphoma [22]. In 1996, breast cancer accounted for 12.8% of all malignant neoplasms admissions at the Korle Bu Teaching Hospital [23]. The increase is even further evident when Clegg-Lamptey et al. reported in 2009 that breast cancer now accounts for about 16% of all cancers, being the commonest female cancer in Ghana [24]. Currently, epidemiologic data on breast cancer is inadequate as most studies are based on clinicopathological characteristics. Ghana also lacks a comprehensive breast cancer control policy and treatment guidelines. Treatment is mainly by surgery, chemotherapy, and radiotherapy in few hospitals which are located in urban centres further creating geographical barriers to accessibility for most Ghanaian women [25]. Furthermore, financial barriers also exist as cost coverage by the National Health Insurance (NHIS) is not the best. Despite the lack of resources and infrastructure, few clinical studies from Ghana and other sub-Saharan communities support the general characterizations reported including severe with unfavourable prognostic features [14], higher prevalence of triple negative breast cancer [17, 26–28], and absconding during treatment [29], but all these do not fully explain the pattern of breast cancer observed in Ghana. Since breast cancer is a complex and heterogeneous disease with ethnic and social variations [30], it is important that each community or population have accurate scientific data that defines the characteristics of the disease among its people so as to determine the most suitable method to control the disease and limit its mortality [31]. It is thus essential to determine and establish the pattern of breast cancer distribution in Ghana taking into consideration factors such as sociodemographic, socioeconomic, aetiological, ethnic, and racial variations. This study was, therefore, conducted in five regions of Ghana to answer three pertinent questions. (i) What is the pattern and incidence of breast cancer in Ghana? (ii) Will the result justify and recommend a mass screening exercise? And (iii) will the result require us to modify our approach to screening campaigns? Our findings we believe may provide the information policy makers need to formulate breast cancer control policy and treatment guidelines in Ghana.

## 2. Methods

### 2.1. Study Area and Design

This is a cross-sectional study in five regions of Ghana: Greater Accra, Volta, Ashanti, Western, and Brong Ahafo. The other five regions not included are Upper East, Upper West, Northern, Eastern, and Central. Region selection was based on (i) population density and accessibility, (ii) willingness of participants, (iii) limited resources, and especially (iv) proximity to treatment centres where suspicious cases could be referred to for further evaluation and treatment. Thus even though Upper East, Upper West, and Northern Regions occupy about 40.9% of the size of Ghana, the comparatively low population density scattered in clusters made accessibility and proximity to the few health facilities very difficult. Characteristics in Eastern and Central Regions are similar to those of Ashanti and Western Regions, respectively, so results in these two larger population dense regions can be extrapolated and representative.

### 2.2. Ethical Considerations

The study originated from the Ministry of Health and Ministry of Women and Children, Ghana, and had gone through and had been approved by the Ministries' Review Boards. Mammocare was granted authority to conduct the study and was required to organize an awareness talk, explain all procedures, and respond to participant's questions before the screening process. Discussions included the harmless procedural process, no coercion and ability to withdraw at one's own will, no invasion of privacy or involvement of deception, and absence of monetary rewards. After all these, only willing participants who picked and completed a consent form were included in the study. Members of the medical team that performed the clinical breast examination (screening) were in good standing with their respective boards at the time of the examination.

### 2.3. Selection of Representative Participants and Data Collection

Awareness creation and detailed explanation of procedures were launched and conducted in churches, mosques, schools, offices, and market places within the five selected regions between January 2007 and November 2008. Willing female participants ranging from 15 years to 74 years underwent clinical breast examination. As part of the screening, participants were guided to respond to questionnaires which captured information such as age, menarche, parity, family history of breast cancer, knowledge level, and perception of breast cancer and its symptoms. All participants with clinically palpable breast lumps were counselled by the medical team and then referred to the district or regional hospitals for confirmation and appropriate treatment. Even though the scope of this study was limited to patterns and incidence, follow-ups were made on the study participants with suspected cases. Applying the Raosoft sample size calculator approach using an incidence of 1% breast abnormality in the population and an absolute precision/confidence level of 0.5% with a design effect of 1.0 for random sample, a total sample size of 3000 women can be used as a true representation of the population in the five regions. In achieving our target number, we did our best to cover most districts in the regions in order to capture a wider distribution of the population.

### 2.4. Confirmation of Palpable Masses

All cases of suspicious palpable lumps were referred to the Regional Health Facilities. Here procedures such as mammography or ultrasonography were applied depending on age. This was followed by fine needle aspiration cytology and tissue histopathology for confirmation of the palpable masses as benign or cancerous.

### 2.5. Data Analysis

Two levels of data analyses were conducted on the acceptable questionnaires. At the first level, univariate analysis was performed to provide a summary and description of the variables considered in the study. Then, descriptive bivariate associations were used in the second level of analyses to explore the relationship between each of the background characteristics of participants and the main outcome variable (results of screening). Here, Pearson's Chi-squared test was applied as the main test of statistical significance at *P* < 0.005. The results from Pearson's Chi-squared test were used for inferential analysis.

## 3. Results

**Figure 1:**
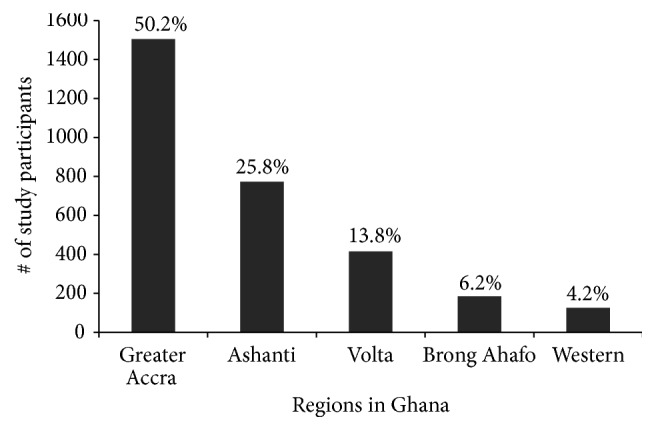
Population distribution of study participants by region. The 3000 female participants in the study were selected based on population density of the region, willingness of participants, and accessibility to treatment facilities for referrals. Percentages shown here were calculated as a fraction of participants in every region over total number of participants recruited.

This study involved 3000 randomly selected female participants and Figure 1 shows the total population distribution per region. About 75% of the study participants were recruited from Ashanti and Greater Accra Regions due to the high population density and accessibility to health facilities in these two regions. Volta Region took half of the remaining 25% followed by Brong Ahafo and Western Regions. Despite fewer numbers in some regions, we involved participants from most districts in order to have a reasonable representation of the general population in the region.

**Figure 2:**
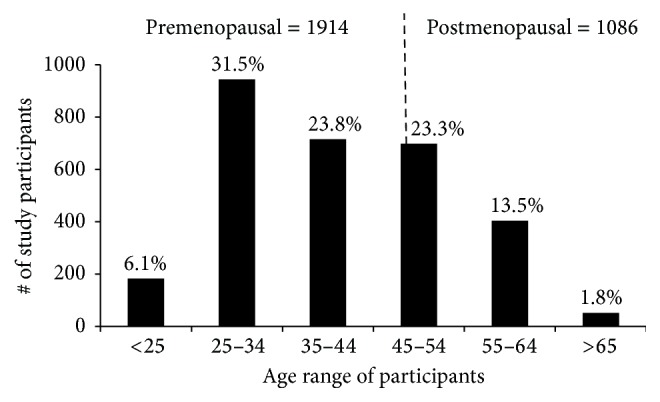
Pre- and postmenopausal classification of study participants. Study participants were categorized as premenopausal or postmenopausal based on the calculated menopause age of 46.6 years (broken line). Seventy-one (71) participants corresponding to 2.36% overall were within the 45–46.6-year group. The number of participants within each age group is shown in Table 1. Percentages shown for each age group were calculated as a fraction of participants within that group over the total participants recruited.

**Figure 3:**
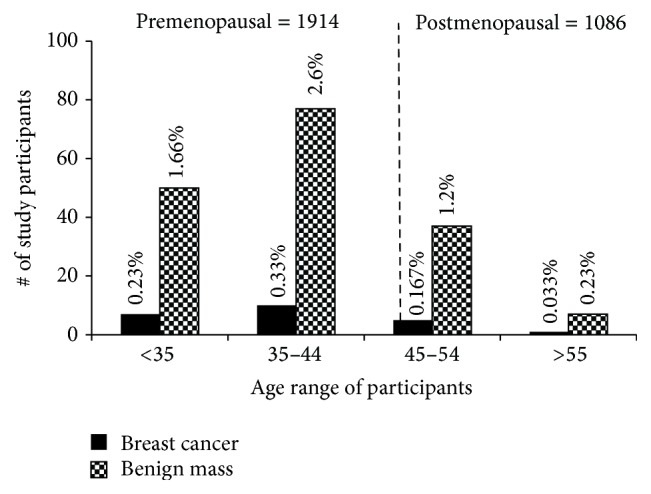
Incidence of breast cancer and breast mass in pre- and postmenopausal participants. Incidence of breast cancer and breast mass is shown in the various age groups in pre- and postmenopausal participants. Notably, most of the participants diagnosed with breast cancer were premenopausal with a mean age of 38 years and seven (7) participants diagnosed were below 35 years. A single benign mass case was observed in the 71 participants between 45 and 46.6 years. More details including histological classifications of the cases are shown in Table 1. Percentages shown for each age group were calculated based on number of breast disease cases diagnosed in each group over total number of participants. *P* < 0.005.

Figure 2 shows the ages of the participants grouped into premenopausal (1914 participants) and postmenopausal (1086 participants) using the average calculated menopausal age of 46.6 years in this study. For ease of grouping, the ages were rounded up to the nearest whole number and the percentage values in this figure and subsequent figures are calculated based on the total number of 3000 participants. Two thousand, three hundred and fifty eight (2358) of the participants (78.6%) were within the 25–54-year age group (Table 1), where cancer cases are most likely to occur. Previous reports have set the average age at which breast cancers are observed in the Black female population including especially women in sub-Saharan Africa to be about 40 years. We decided to ascertain whether such generalized statements are still applicable in Ghana. Clinical breast examination of the 1914 premenopausal women shows 145 (4.83%) participants having clinically palpable breast lumps. Of these, 17 (0.56%) were confirmed as having breast cancer and 128 (4.26%) as benign masses. Of the 17 confirmed cases, 14 were invasive ductal carcinomas and the remaining 3 were invasive lobular carcinomas (Figure 3; Table 1). Comparatively, within the 1086 postmenopausal women, 6 (0.2%) and 43 (1.43%) were confirmed to have breast cancer and benign masses, respectively. Four (4) of these 6 cases were invasive ductal carcinoma, one (1) was invasive lobular carcinoma, and one (1) was in situ carcinoma (Figure 3; Table 1). Assessing premenopausal incidence within the various age groups, the most breast cancer and benign masses cases of 10 (0.33%) and 78 (2.6%), respectively, are observed within participants of the 35–46.6-year age group (Table 1). Surprisingly, there is significant observation of breast cancer and benign masses in participants below 35 years, 7 (0.23%) and 50 (1.66%), respectively (Figure 3; Table 1), suggesting an increasing possibility of a downshift of breast cancer with respect to age. In Western countries, the postmenopausal group has the highest cases of breast cancer. However, this may not be so in Ghana because we observed 38 years as the average mean age for breast cancer and only 6 out of the 23 cancer cases were postmenopausal (Figure 3; Table 1). We did not see any breast cancer cases above 65 years and this could be due to only 52 participants (Figures 2 and 3 and Table 1). This is the first time a study in sub-Saharan African country is reporting an increasing percentage of breast cancer cases in the below 35-year age group, 10 years earlier than previously reported, suggesting an increasing risk in younger Ghanaian women.

With 145 palpable masses, the remaining 1769 premenopausal participants group under Aberrations of Normal Development and Involution (ANDI): 455; Abscesses, Infections, and Mastitis (AIMS): 169; and No Abnormalities Detected (NAD): 1145 (Figure 8). Similarly, for the remaining 1037 postmenopausal participants, the groupings are 388, 43, and 606 for ANDI, AIMS, and NAD, respectively (Figure 8). For this study, our main focus was on descriptive statistics of clinically palpable masses so we concentrated less on ANDI and AIMS.

**Figure 4:**
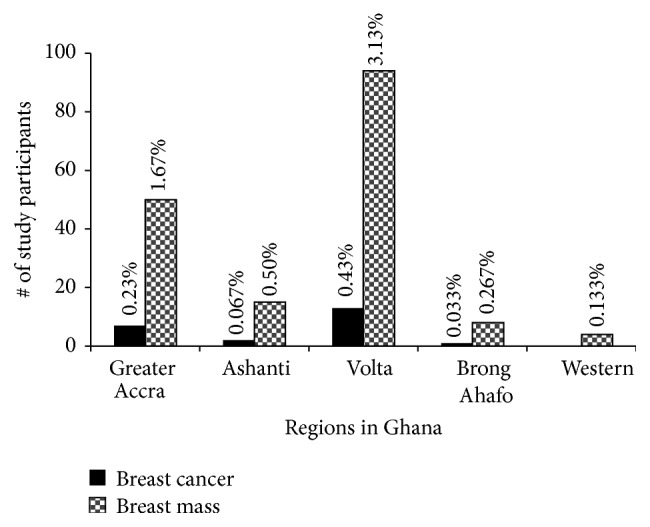
Regional incidence of breast cancer and breast mass in study participants. Incidence and distribution of breast cancer in the five regions under study are shown. Notably, Volta Region had the highest case burden followed by Greater Accra Region. Histological classifications per region are shown in Table 2. The percentages were calculated based on the total number of diagnosed cases per region over total participants recruited. *P* < 0.005.

We next decided to ascertain the regional distribution of breast cancer and benign masses. In Figure 4, the regional incidence of breast cancer ranges from zero in the Western Region to a high of 0.43% in the Volta Region, which incidentally also had the highest benign cases (3.13%). The incidence of 0.43% in Volta Region is puzzling as nothing peculiar has been reported of Volta Region. Volta Region also had the highest number of cancer cases for both ductal and lobular invasive cancers followed by Greater Accra Region. The only in situ case was in the Brong Ahafo Region (Table 2). The low incidence of the breast disease in the Brong Ahafo and Western Regions could be attributed to the small sample number; hence a second larger study may be warranted.

**Figure 5:**
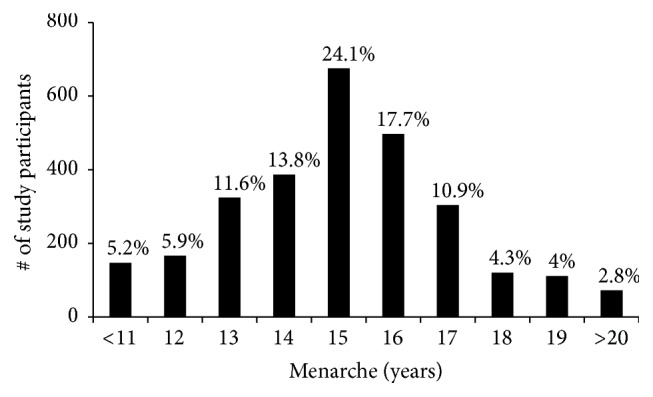
Age specific distribution of menarche among the study participants. The average female in Ghana experiences menarche at 15 years. Participants below 15 years were classified as early menarche and above 15 years were classified as late menarche.

**Figure 6:**
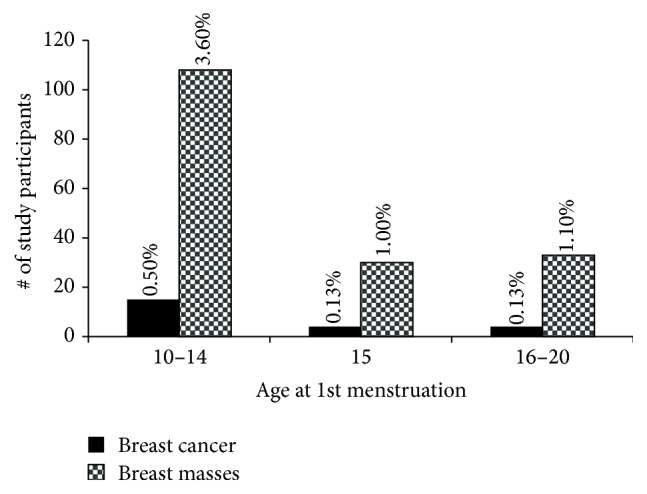
Incidence of breast cancer and breast mass relative to menarche. Study participants assessed for relationship between onset of menstruation and breast cancer are shown. Notably, participants who experienced early menarche were at a greater risk of breast disease. Table 3 further shows the corresponding histological classifications. *P* < 0.005.

A common risk factor for breast cancer is age at first menstruation (menarche). The earlier you menstruate the higher the risk for breast cancer is.  Figure 5 shows the average age of menarche among study participants with the peak age being 15 years (24%). Thus 11 to 14 years [1024 participants (36.5%)] will be considered early and greater than 15 years [1104 participants (39.39%)] will be considered late. In Figure 6 and Table 3, 15 (0.50%) and 108 (3.6%) participants in the early menarche group have breast cancer and breast masses, respectively, compared to 4 (0.13%) and 33 (1.1%) participants in the late menarche group. Of the participants who had menarche at 15 years, 4 (0.13%) and 30 (1.0%) were observed to have breast cancer and breast masses, respectively. Of the 15 confirmed cases in the early menstrual group, 12 were invasive ductal, 2 invasive lobular, and 1 in situ carcinoma. The other two groups had 4 cases with 3 ductal and 1 lobular each (Table 3). We do not know if there is any relationship between early/late menstruations and the type of cancer. These results confirm previous reports that early menarche increases the risks of breast cancer and this assertion may be applicable to sub-Saharan Africans as well.

Other known protective factors such as the age at first pregnancy and the number of pregnancies were also examined. We report 0.07%, 0.23%, 0.43%, and 0.03% breast cancer for participants who had their first child at <19, 20–24, 25–29, and 30–34, respectively (see Supplementary Figure 1 in Supplementary Material available online at http://dx.doi.org/10.1155/2016/3645308). This result may support previous reports that early pregnancies reduce the risk of breast cancer, though the 0.03% observed for participants who had their first child between 30–34 years digresses from expectation. Results on whether multiparity offers protection were inconclusive (not shown) and it also did not matter whether you were married or single (not shown) even though some women in sub-Saharan Africa believe that marriage offers some protection.

**Figure 7:**
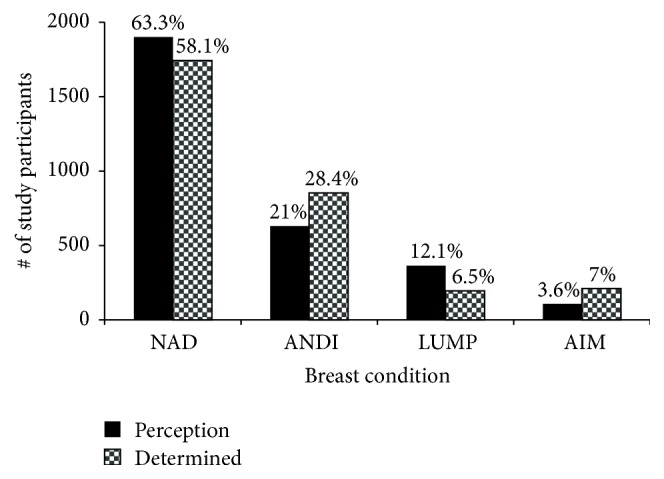
Comparison of perception and determination of breast anomalies among study participants. Participants were asked to examine and report any adverse findings in the breasts (perception) and the results were compared with clinical breast examination by qualified personnel (determined). The differences between the perceived and the determined suggest intensification of the campaign message.

Breast cancer awareness campaigns are on the increase in Ghana, though not quite close to the Western world. We wanted to ascertain the level of awareness and response to these campaigns so part of the questionnaire captured whether study participants perceived the presence of lumps and/or detected any abnormalities in their breast through simple observation or self-breast examination. We then compared their results with results determined by the clinical breast examination (CBE) team.  Figure 7 shows that the message of the awareness campaigns may be reaching the population. For example, 21% of study participants reported the presence of ANDI on their breasts and the clinical team confirmed so in 28.4% of cases. Previous observations have shown little knowledge of ANDI (undocumented). Even though 12.1% of participants reported presence of what they thought to be suspicious lumps, the CBE team actually confirmed so in 6.5% of the participants. The significant differences between perception and reality will be difficult to ascertain due to the fact that this was not an intervention study. This is elaborated further in Discussion.

**Figure 8:**
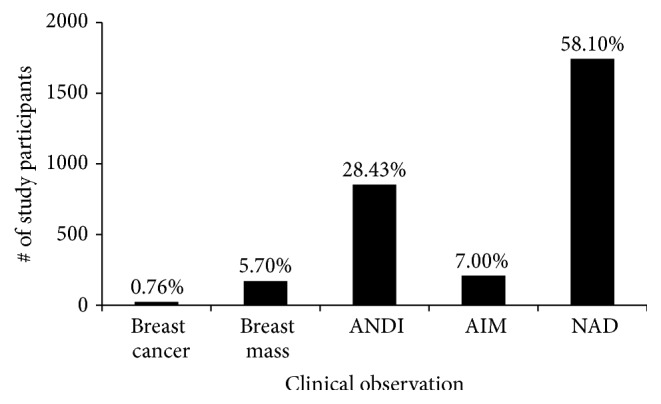
Overall summary of incidence of breast lesions. Various percentages of breast lesion incidences are shown. ANDI: Aberrations of Normal Development and Involution; AIM: Abscesses, Infections, and Mastitis; and NAD: No Abnormalities Detected. The incidence of breast cancer in Ghana is 0.76%, which is predicted to increase with time.

Figure 8 summarizes our overall findings. Notably, the incidence breast cancer rate in Ghana from this study is about 0.76% and that for breast mass is about 5.7%. However, our major findings of peak breast cancer cases at 38 years (2 years lower than proposed) and an increasing number of cases below 35 years suggest a possible downward shift of breast cancer burden in Ghanaian women.

## 4. Discussion

Our aim was to accurately assess the incidence and pattern of breast cancer distribution in Ghana to enhance detection, diagnosis, and treatment. Breast cancer is complex and has been associated with ethnic origins, so it is important to ascertain any peculiar characteristics of the disease locally, to enable the recommendation of the most suitable control method to reduce mortality. The 2010 Ghana population census report [32] estimates a total of 12,633,978 females in Ghana. The total number of females residing in the five regions where the study took place is 7,988,970. However 39.4% of females are either below 15 years or above 74 years, so the total eligible population considered for the purposes of this study is 4,921,205. The average increase in growth rate from 2000 to 2010 is about 2.4% [32]; hence the eligible population of 4,921,205 selected exceeds the expected population in 2008. Using the above population number and applying the Raosoft sample size calculator approach, the sample size of 3000 participants aged between 15 and 74 years can be used as a true representation of the population in the five regions under study.

Even though the 2010 census estimates [32] show that Ashanti Region (AR) has a greater female population than Greater Accra Region (GAR) [(2,464,328 to 2,071,829, resp.)], the population density in GAR (1236/Km^2^) due to its smaller size far exceeds the other four regions combined (457/Km^2^). Together, with accessibility to willing participants and proximity to treatment centres explains why most of the participants were from GAR (Figure 1).

Menopause occurs naturally in women between 45 and 55 years, and the average age in the developed world is 51.4 years [33]. Another study that included data from 26 different countries reports a lower average age of 49.24 years [34]. Agyei-Frempong et al., in 2008, estimated the average menopausal age in Ghana to be 47.77 years [35]; however we calculated an average of 46.6 years in this study. We thus grouped the study participants into premenopausal and postmenopausal based on this age (Figure 2). Previous reports [13, 28, 30, 36–39] predict 40 years as the peak age for breast cancer in sub-Saharan Africa, and our results showing the highest burden in the 35–46.6-year group support these reports. Most importantly, we also show increasing cases of breast cancer and benign masses (30% in both cases) in participants below 35 years indicating that younger Ghanaians are more at risk than previously reported. In advanced countries breast cancer is observed more in the 55- to 64-year group [13, 36, 40, 41]. However, our results showing low breast cancer incidence in age groups 45–54 and 55–64, respectively, suggest that the probability of the Ghanaian woman getting breast cancer is very high premenopausally. Thus our target group for awareness campaigns should be the second cycle schools (high schools) and breast screening should be started much earlier preferably from the early 20s, which will also require a new mode of screening. Furthermore, with unique diseases such as breast cancer, we should also encourage local research as our results suggest a downward shift in age and possibly the need for the revision of guidelines for breast screening at least in Ghanaian women.

Breast cancer is categorized broadly into in situ and invasive carcinomas. In situ carcinoma can be further subgrouped into ductal carcinoma in situ and lobular carcinoma in situ and the former is more common. Invasive carcinomas also have subgrouping and the two common ones are invasive ductal carcinoma and invasive lobular carcinoma, and again the ductal is more common. In our case, we confirmed eighteen (18) of the cases as invasive ductal carcinoma, four (4) as invasive lobular ductal carcinoma, and one (1) as in situ carcinoma. Our results are in conformity with other reports where 70–80% of all invasive lesions are invasive ductal carcinomas [42, 43] and about 15% are invasive lobular carcinomas [42]. We could not ascertain if there are any correlations between age, place of abode, early menarche, and the type of breast cancer. Even though it is recommended that molecular marker status be determined for all invasive carcinomas, this was not done here due to lack of resources. Furthermore, since rapid treatment mostly in the form of surgery is required due to late presentation of cases, grouping them as invasive or in situ enables treatment to start.

The low incidence of breast cancer in sub-Saharan Africa has been attributed to factors such as late menarche, early child-bearing age, high parity with prolonged lactating, and irregular menses. As sub-Saharan countries shift from low to lower middle income, significant changes such as early menarche, early childbirth, reduced parity, and short lactating periods are on the increase and this may be the reason why we are seeing the increasing incidence in comparatively younger women, though other factors such as diet, socioeconomic status, and family history cannot be ruled out.

Assessing the regional distribution, the 0.43% of breast cancer observed in the Volta Region (56% of all the breast cancer cases) is on the high side and very puzzling considering that the population sampled in Volta Region is about a quarter of Greater Accra Region. Our questionnaire did not capture migration patterns, lifestyle choices, and type of stable food preparation and intake, so we cannot for now give any tangible reason for such incidence. Despite that, we find it to be way out of the norm and therefore recommend performing genomic surveillance and rescreening in the Volta Region with more participants using questionnaires that capture more information.

Our results are in line with previous reports asserting early menarche as a risk factor for breast cancer [13, 36, 40, 44]. Setting 11 to 14 years (1024 participants) and 16 to 19 years (1032 participants) as early and late menarche, respectively, we can say that late menarche offered some form of protection (0.13% compared to 0.5%). This is in contrast to a study conducted in Nigeria which could not pinpoint menarche as a risk or protective factor [45]. However, several studies [13, 36, 40, 45] have shown that reducing length of exposure to oestrogen as in late menarche and early childbirth may account for the protection.

Our analysis of whether early childbirth offered protection showed positive correlation with known literature. However, a low percentage (0.03%) of breast cancer was observed for women who had their first child between 30 and 34 years. We are yet to find an explanation for this observation. Other results such as multiparity and long lactating times are not shown, but multivariate analysis carried out shows that neither long lactating times nor multiparity confirmed or refuted previous reports that they reduce the risk of breast cancer. Rosenberg et al. in 2002 reported that women in South Africa with childbirth at age 30 years or older had a twofold increase in breast cancer risk compared to women who had their first child at 16 years or younger [46]. Coogan et al. (1999) confirmed Rosenberg's assertion, in addition to their reporting that breast carcinoma risk was not found related to the duration of breast-feeding the first child, the number of children breast-fed, or the patient's age at first lactation [47]. With such conflicting data in Africa on the protective and nonprotective factors, perhaps a multinational collaborative study taking into account lifestyle, postbirth practices, culture, diet, and socioeconomic status, among others, is warranted to resolve these differences in results.

Both government and nongovernmental organizations in Ghana have been carrying out public education campaigns to create breast cancer awareness. To determine the impact of these campaigns, study participants were asked to perform self-breast examination (SBE) on their breasts and report suspicious lumps and/or any abnormalities detected. The team then conducted thorough clinical breast examination and the two results were compared (Figure 7). Though there are differences between the perceived and the determined, it is not easy to ascertain the significant relationship between the values. It will have been possible if we had pregathered these 3000 participants, trained them on SBE, and then assessed the results of our training. Since this was not an intervention study and there was no previous training from different awareness groups, we are reporting it “as is.” In any case, the fact that more women perceived the presence of lumps than was really detected is a good sign if and only if they will follow up for further analysis. It is thus essential for policy makers to increase support for breast cancer awareness as the message seems to be getting to the people who need it most.

## 5. Conclusion

In conclusion, this project was conducted to answer three pertinent questions. (i) What is the pattern and incidence of breast cancer in Ghana? (ii) Will the result justify and recommend mass screening of Ghanaian women? And (iii) will the result require us to modify our approach to screening campaigns? Our results reveal a breast cancer incidence of 0.76% and an increasing incidence in women below 35 years. So, on the side of caution, we propose a review of the guidelines, such that breast cancer awareness campaigns should start in the senior high schools and breast screening for Ghanaian ladies should start from 21 years onwards. The result also justifies mass screening in Ghana and the establishment of a national breast cancer registry to formulate a control and treatment policy in Ghana. Since the number of breast cancer cases will increase in the coming years, such controlled policies will enable us to use our scarce resources to improve quality of service and fund interventions that provide value for money.

## Supplementary Material

2548 of the 3000 participants who partook in the screening exercise had been pregnant before. The questionnaire captured the age at first pregnancy and the results were then categorized under the following groupings: <19 years (284), 20–24 years (958), 25–29 years (849), 30–34 years (427), and >35 years (30). The ages were rounded up to the next highest whole number and the incidence of breast cancer and breast mass for each age group ascertained.

## Figures and Tables

**Table 1:** Histological classification of breast cancer in relation to various age groupings. All histological classifications are based on examination of tissue specimens. Most of the cases were invasive ductal carcinomas.

Age group	# of participants	Breast cancer cases	Benign mass cases	Invasive ductal carcinoma	Invasive lobular carcinoma	In situ carcinoma
<25	183	1	5	1	0	0
25–34	945	6	45	5	1	0
35–44	715	10	77	8	2	0
45–46.6	71	0	1	0	0	0
46.7–54	627	5	36	4	1	0
55–64	406	1	7	0	0	1
>65	53	0	0	0	0	0

Totals	3000	23	171	18	4	1

**Table 2:** Histological classification of breast cancer in the selected regions in Ghana. Volta Region unexpectedly had the highest burden.

Region	Invasive ductal carcinoma	Invasive lobular carcinoma	In situ carcinoma	Benign masses
Greater Accra	6	1	0	50
Ashanti	1	1	0	15
Volta	11	2	0	94
Brong Ahafo	0	0	1	8
Western	0	0	0	4

Totals	18	4	1	171

**Table 3:** Relationship between breast cancer histology and onset of menstruation. Early menarche is an increased risk factor.

Menarche	Invasive ductal carcinoma	Invasive lobular carcinoma	In situ carcinoma	Benign masses
10–14	12	2	1	108
15	3	1	0	30
16–20	3	1	0	33

Totals	18	4	1	171
