# Supplementary figures and images for: Pattern of Breast Cancer Distribution in Ghana: A Survey to Enhance Early Detection, Diagnosis, and Treatment
Source: Int J Breast Cancer. 2016 Aug 18;2016:3645308. doi: 10.1155/2016/3645308 (PMC5007313; doi:10.1155/2016/3645308)

Supplementary Figure 1

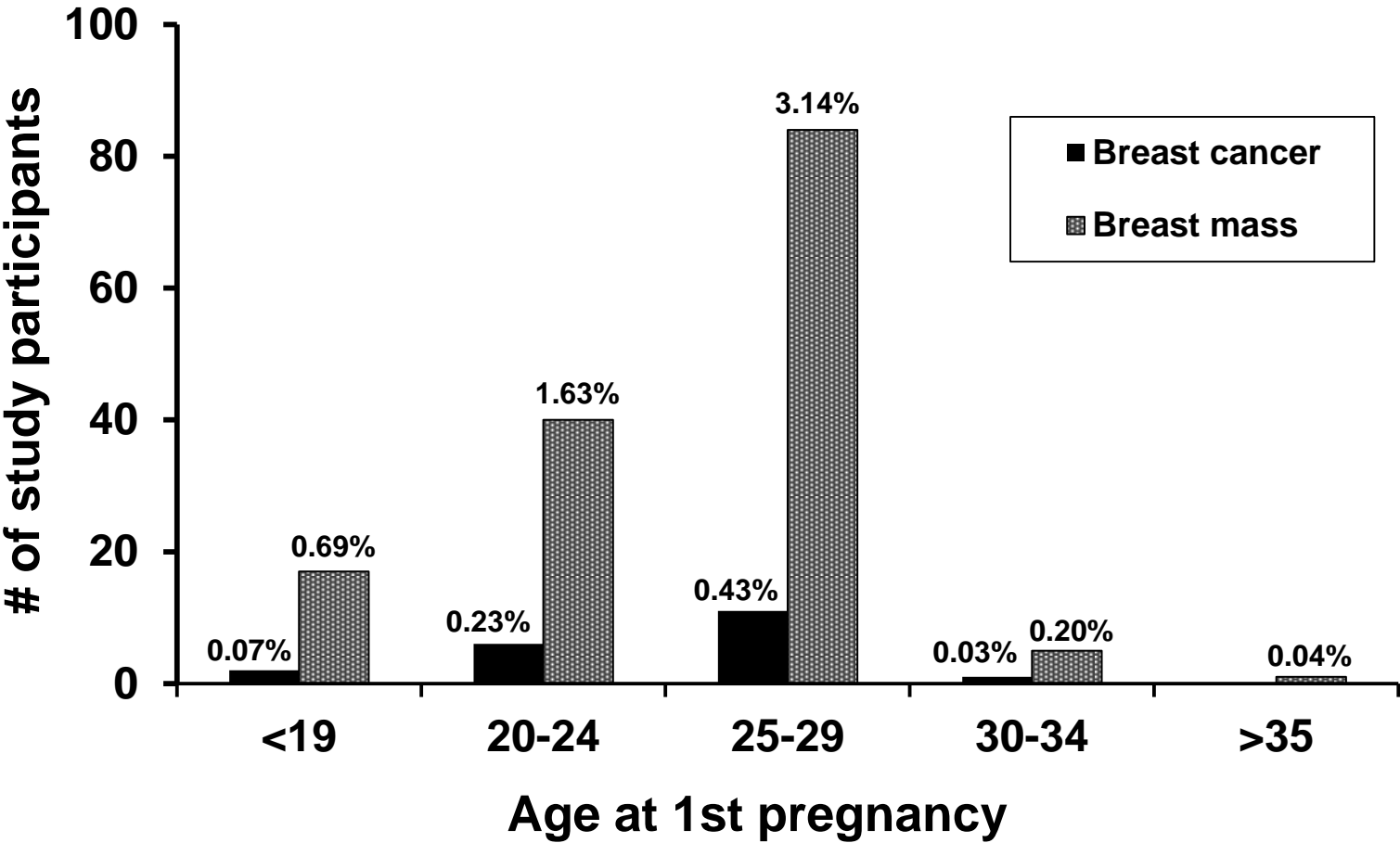

Supplement: Supplementary file 1 — 2548 of the 3000 participants who partook in the screening exercise had been pregnant before. The questionnaire captured the age at first pregnancy and the results were then categorized under the following groupings: <19 years (284), 20–24 years (958), 25–29 years (849), 30–34 years (427), and >35 years (30). The ages were rounded up to the next highest whole number and the incidence of breast cancer and breast mass for each age group ascertained. [file 3645308.f1.pdf]
